# Engineering Drought-Responsive ABA Signaling: Linking Physiological Stress Cues to Metal–Organic Framework-Mediated ABA Delivery and SnRK2 Activation

**DOI:** 10.3390/plants15203142

**Published:** 2026-10-14

**Authors:** Muhammad Faheem Jan, Muhammad Ali Shah, Imran Azeem, Haseeb Ahmad, Rehmat Ullah, Waqas Liaqat, Muhammad Aasim, Muhammad Tanveer Altaf

**Affiliations:** 1 Guangxi Key Laboratory of Agro-Environment and Agro-Products Safety, College of Agriculture, Guangxi University, Nanning 530004, China; mfaheemjan366@gmail.com (M.F.J.); haseebagr63@gmail.com (H.A.); 2 School of Breeding and Multiplication (Sanya Institute of Breeding and Multiplication), Hainan University, Sanya 572025, China; alik98741@gmail.com (M.A.S.); imranazeem19@gmail.com (I.A.); 3 Key Laboratory of Crop Genetic Breeding and Germplasm Innovation, Guangxi Key Laboratory of Agro-Environment and Agri-Products Safety, College of Agriculture, Guangxi University, Nanning 530004, China; 4 Department of Field Crops, Faculty of Agriculture, Recep Tayyip Erdoğan University, Pazar, Rize 53300, Türkiye; muhammadtanveer.altaf@erdogan.edu.tr; 5 Department of Precision Agriculture and Agricultural Robots, Faculty of Agricultural Sciences and Technology, Sivas University of Science and Technology, Sivas 58000, Türkiye

**Keywords:** drought stress, redox homeostasis, reactive oxygen species, SnRK2 kinases, metal–organic framework, stomatal regulation, stimulus-responsive nano delivery

## Abstract

Drought induces coordinated physiological, biochemical, and molecular responses that extend beyond changes in phytohormone concentrations. Rapid alterations in reactive oxygen species (ROS), apoplastic pH, Ca^2+^ and ionic dynamics, tissue hydration, and cellular redox status provide spatial and temporal information on developing water deficit and regulate stomatal behavior, photosynthesis, water relations, and stress-responsive gene expression. This review explores whether endogenous drought-associated signals can serve as biological triggers for stimulus-responsive metal–organic frameworks (MOFs) designed to regulate abscisic acid (ABA) delivery. We integrate drought-induced changes in redox state, apoplastic pH, ionic composition, and hydration with MOF transformation, host–guest interactions, ABA release, uptake, transport, metabolism, and engagement of the PYR/PYL/RCAR–PP2C–SnRK2 signaling pathway. We propose a framework in which responsive MOFs generate a temporally controlled ABA pulse that follows drought progression, activates protective signaling during water limitation, and attenuates after rewatering to facilitate recovery. Particular emphasis is placed on ROS-Ca^2+^ crosstalk, SnRK2/OST1-dependent guard-cell regulation, hydraulic adjustment, photosynthetic protection, and recovery dynamics. Current studies demonstrate MOF-mediated phytohormone protection, controlled release, and drought-related physiological improvement. Current advances have established the feasibility of MOF-based ABA protection and controlled delivery; however, the integration of drought-responsive carrier activation with receptor-accessible ABA delivery and PYR/PYL/RCAR–PP2C–SnRK2 pathway validation remains an emerging research frontier. We therefore outline a claim-matched validation framework integrating spatial drought-signal analysis, isotope-resolved ABA tracing, carrier localization, phosphorylation-based pathway assays, drought–rewatering experiments, and physiological assessment. This integration of plant stress physiology with responsive nanomaterials provides a conceptual basis for developing more precise ABA delivery strategies for crop resilience under increasingly variable water availability.

## 1. Introduction

For crops, drought is not a fixed state but a dynamic stress that unfolds across space and time. Soil water potential declines progressively during soil drying and can increase rapidly following rainfall or irrigation, whereas variation in atmospheric vapor-pressure deficit regulates stomatal conductance, transpiration and photosynthesis [1,2]. Roots simultaneously experience microscale heterogeneity in water availability and preferentially position lateral branches toward wetter soil domains [3]. The timing of rewatering is especially important during reproductive development because water deficits at flowering and early seed set can impair pollen function and seed formation, and these reproductive losses may not be fully recovered after water availability is restored [4,5]. Plants must therefore activate water-conserving responses rapidly enough to preserve hydraulic integrity, yet attenuate them after rewatering to restore stomatal conductance and carbon assimilation [6,7]. Abscisic acid (ABA) is central to this coordination. It induces stomatal closure, adjusts root and leaf hydraulic conductance, coordinates osmotic and antioxidant defenses, and reprograms gene expression through the conserved PYR/PYL/RCAR–clade A PP2C–SnRK2 signaling module [8,9]. ABA also influences seed development, root architecture, flowering, and yield formation, making the timing, duration, and tissue distribution of the signal as important as bulk hormone abundance [10]. Live biosensors have further revealed that stress-induced ABA accumulation and transport are strongly tissue-dependent, reinforcing the need to consider ABA as a spatially and temporally structured signal rather than a uniform whole-plant concentration [11].

Exogenous ABA can improve plant water status and reduce drought injury, but conventional application cannot reproduce the spatial and temporal precision of endogenous signaling. Free ABA is photolabile and, following foliar application, is also vulnerable to wash-off and off-target loss. Once absorbed, CYP707A enzymes may hydroxylate it, conjugate it to an ABA-glucose ester (ABA-GE), redistribute it through vascular tissues, or sequester it in cellular compartments [12,13]. These processes uncouple the applied dose from the bioactive ABA reaching target cells, while timing further constrains efficacy. ABA applied prematurely may dissipate before drought, whereas persistent exposure after rewatering can sustain stomatal closure, restrict CO_2_ assimilation, and delay growth recovery. The prolonged antitranspirant activity of high-affinity ABA–receptor agonists demonstrates how ligand potency and persistence determine response duration [14]. Thus, maximizing ABA loading or cumulative release is not an adequate design objective. Instead, the goal should be to generate a transient increase in bioavailable ABA whose onset, magnitude, tissue distribution, and termination track the trajectory of water deficit. Here, an “ABA pulse” denotes such a bounded increase in bioavailable hormone and does not imply that endogenous ABA necessarily follows an oscillatory pattern.

Metal–organic frameworks (MOFs) offer a modular platform for controlling ABA protection and release. These crystalline coordination networks comprise metal ions or polynuclear clusters connected by multitopic organic ligands, generating ordered pores whose topology, dimensions, chemical microenvironment and stability can be rationally adjusted through node and linker selection, multivariate functionalization and defect engineering [15,16]. Control over crystal size, surface chemistry and supramolecular gating provides additional leverage over dispersion, cargo retention, framework degradation and stimulus-dependent release [17,18]. MOFs have been investigated as carriers for fertilizers, pesticides, biomolecules and plant growth regulators [19,20]. Most directly, ABA encapsulation in MIL-100(Fe) reduced UV-induced photodegradation and accelerated release under neutral-to-alkaline relative to acidic conditions, consistent with a design intended to exploit drought-associated apoplastic alkalinization. The formulation also improved drought-related physiological traits in cotton seedlings [21]. This study established carrier-level feasibility but did not demonstrate stress-triggered release in plants, resolve carrier uptake or tissue-specific ABA exposure, verify activation of the PYR/PYL–PP2C–SnRK2 pathway, or assess post-drought recovery and reproductive yield.

Agricultural MOF reviews primarily address synthesis, loading, release kinetics, crop protection and environmental performance [22,23,24], whereas ABA–SnRK2 reviews examine hormone metabolism, transport and signaling without integrating responsive material delivery [25,26]. This gap is biologically important because MOF-released ABA would enter a drought-activated network in which B2–B4 RAF kinases provide ABA-dependent and ABA-independent inputs to SnRK2 activation [27,28]. RAF-initiated phosphorylation and subsequent SnRK2 transphosphorylation can further amplify kinase activation [29]. Thus, the SnRK2 activation threshold is not defined by a universal ABA concentration but by receptor abundance, PP2C activity, RAF input, tissue and developmental context, and stress history. A mechanistic framework connecting MOF release kinetics with this dynamic signaling architecture remains lacking.

**Figure 1:**
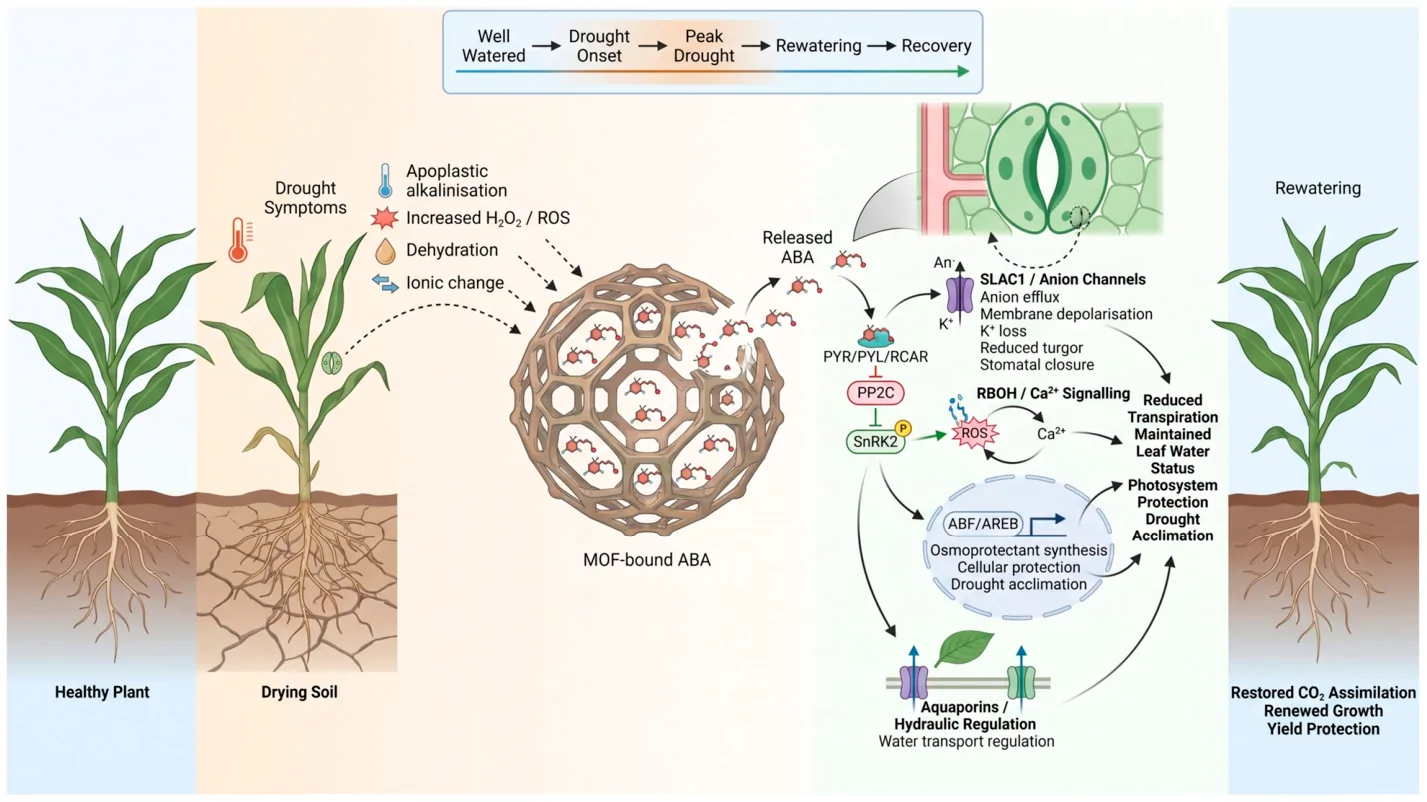
Integrated drought–MOF–ABA–SnRK2 signaling framework. Drought-associated alkalinization, reactive oxygen species, dehydration, and ionic shifts trigger MOF transformation and controlled ABA release. Released ABA is perceived by PYR/PYL/RCAR receptors, which inhibit clade A PP2Cs and permit activation of ABA-responsive SnRK2 kinases. SnRK2-dependent regulation of SLAC1, RBOH proteins, aquaporins, and ABF/AREB transcription factors links stomatal closure with hydraulic adjustment, osmoprotection, and stress-responsive transcription. Signal attenuation after rewatering supports stomatal reopening, carbon assimilation, and growth recovery. Solid arrows denote established biological relationships; dashed arrows denote proposed or incompletely validated MOF-mediated connections.

We address five linked questions: (1) which drought-associated plant or rhizosphere cues can trigger ABA release under field-relevant conditions; (2) how MOF composition and release kinetics should be matched to PYR/PYL/RCAR–PP2C–SnRK2 signaling; (3) how uptake, transport, carrier transformation and ABA metabolism determine target-cell exposure; (4) which signaling components are conserved across crops and which crop-specific traits require formulation adjustment; and (5) what evidence is needed to establish triggered release, pathway engagement, post-stress recovery, agronomic benefit and environmental safety. Our central proposition is that MOF-mediated ABA delivery should be evaluated as a dynamic signaling intervention that links drought-responsive release to SnRK2 activation, recovery, and yield formation (Figure 1).

## 2. Constraints on Conventional ABA Delivery

### 2.1. Physicochemical Stability, Uptake and Biological Fate of Exogenous ABA

Conventionally applied ABA is constrained by degradation and inefficient delivery before receptor engagement. Its stability depends on irradiance, temperature, pH, formulation, and residence time. Under 254 nm ultraviolet irradiation in vitro, free ABA degraded more rapidly than ABA encapsulated in MIL-100(Fe), demonstrating photoprotection under controlled conditions but not field stability [21]. Foliar delivery is further limited by droplet retention, wash-off, and cuticular penetration. As a weak acid (pKₐ ≈ 4.8), ABA partitions between membrane-permeable ABA-H and anionic ABA^−^; local pH therefore regulates cellular uptake and apoplastic retention [30]. Soil application introduces additional losses through dilution, sorption, microbial degradation and uneven root exposure. Consequently, the applied dose poorly predicts receptor-accessible ABA [9].

Following uptake, ABA availability is determined by metabolism and transport. CYP707A enzymes catalyze ABA 8′-hydroxylation, producing an unstable intermediate that is converted primarily to phaseic acid [12]. ABA can also be conjugated to glucose as ABA-glucose ester (ABA-GE), which β-glucosidases hydrolyze to regenerate active ABA during stress [31]. Tissue ABA measurements therefore cannot distinguish formulated ABA from endogenous synthesis or ABA-GE hydrolysis. Mechanistic studies should quantify free ABA, ABA-GE and major catabolites, with isotope-labeled ABA used for source resolution. AtABCG25 mediates ABA export, AtABCG40 facilitates uptake, and NPF transporters regulate tissue-specific redistribution [32,33,34]. Formulations targeting stomatal responses must therefore establish ABA delivery and uptake at guard cells rather than infer efficacy from whole-leaf retention.

### 2.2. ABA Dose–Time Dependence and the Survival–Growth Trade-Off

ABA responses are determined by dose, exposure duration, tissue and developmental context. Moderate, transient signaling can conserve water with limited carbon penalties, whereas excessive or prolonged activation restricts stomatal conductance, leaf expansion and reproductive development. Seedling survival therefore does not predict agronomic performance. In rice, SAPK2 regulates grain yield and nitrogen metabolism under reproductive-stage drought, illustrating that SnRK2 functions extend beyond early stress survival [35]. Timing is equally critical: pre-stress application may impose unnecessary growth costs, late application may not prevent hydraulic injury, and persistent ABA after rewatering can delay recovery. In grapevine, stomata remained closed while foliar ABA was elevated, even after leaf water potential and petiole hydraulic function had recovered [36].

ABA-induced stomatal closure conserves water but restricts CO_2_ diffusion, meaning that increased instantaneous water-use efficiency (WUE) may conceal losses in cumulative carbon assimilation. Combined disruption of SnRK2.2, SnRK2.3 and SnRK2.6/OST1 causes strong ABA insensitivity and impairs drought-responsive transcription, confirming that these kinases coordinate stomatal and transcriptional responses [37,38]. An effective carrier should therefore produce a bounded ABA pulse characterized by minimal baseline leakage, drought-triggered release, and rapid attenuation after rewatering. This release profile distinguishes stress-programmed delivery from conventional sustained release.

## 3. MOFs as Programmable ABA Carriers

### 3.1. Framework Chemistry, ABA Loading and Host–Guest Interactions

MOFs are crystalline coordination networks constructed from metal ions or clusters and multidentate organic linkers. Their topology, pore dimensions, internal chemistry and stability can be adjusted through node and linker selection, defect engineering and surface functionalization. Metal–organic frameworks are generally synthesized through coordination-driven self-assembly between inorganic metal ions or metal clusters and multitopic organic ligands. During synthesis, metal centers serve as coordination nodes, whereas organic ligands containing functional groups such as carboxylates, imidazoles, or phosphonates act as bridging linkers that coordinate with metal nodes through metal–ligand coordination bonds, generating ordered crystalline networks with well-defined pore structures [39,40]. The resulting MOF architecture, including pore size, surface chemistry, structural stability, and defect density, is determined by the nature and coordination geometry of the metal nodes, ligand characteristics, solvent system, pH, temperature, and other reaction parameters [41]. ABA can be incorporated into MOF carriers through different loading strategies, including in situ encapsulation during framework formation, post-synthetic impregnation into preformed porous structures, or adsorption within pores and cavities through host–guest interactions [18,41]. The retention and release behavior of ABA are governed by interactions between ABA molecules and the MOF matrix, including hydrogen bonding, hydrophobic interactions, electrostatic interactions, and coordination with accessible metal sites. These interactions can protect ABA from environmental degradation while enabling controlled release through pore diffusion, competitive displacement, framework transformation, or stimulus-responsive gate opening [18,21]. Framework families investigated for biological or agricultural delivery include MIL, UiO, ZIF, PCN and cyclodextrin-derived MOFs. Metal–linker chemistry governs coordination strength, redox activity, hydrolytic stability and degradation, whereas particle size and surface charge influence dispersion, leaf retention and interactions with plant surfaces [22].

Carrier selection must balance ABA loading and release with framework stability and biological activity. MIL-100(Fe) combines large cages, accessible Fe sites and high porosity with demonstrated pH-dependent ABA release. However, released Fe may independently affect nutrition and redox metabolism, confounding ABA-specific responses. UiO frameworks possess strong Zr–oxo-cluster–carboxylate connectivity and high hydrolytic stability, which can reduce premature leakage but prolong environmental persistence. ZIF-8 undergoes acid-promoted dissolution, making its release behavior poorly aligned with drought-associated apoplastic alkalinization. Cu-containing MOFs may provide antimicrobial or catalytic activity but can also promote oxidative injury. The nutritional roles of Fe, Zn and Cu therefore do not establish framework biocompatibility, which depends on dose, dissolution kinetics, particle properties, linker chemistry and exposure duration [42].

ABA may be retained through pore confinement, surface adsorption, hydrogen bonding, electrostatic or hydrophobic interactions, and coordination with accessible metal sites. These interactions determine loading, protection, and release reversibility. Strong binding may stabilize ABA but delay its release, whereas weak surface adsorption can cause premature leakage. Loading content and encapsulation efficiency should therefore be supported by mass balance, desorption analysis, and discrimination between pore-confined and surface-associated ABA. Chemical and stereochemical integrity must also be verified after loading and release. Plant-hormone delivery studies demonstrate how host–guest interactions and supramolecular gates can regulate stimulus-responsive release [18,21].

Synthesis and loading conditions can alter ABA stability, framework defects and batch reproducibility. Physicochemical characterization should include crystallinity, morphology, particle-size distribution, zeta potential in relevant media, porosity before and after loading, spectroscopy, thermal behavior, chromatographic ABA quantification and formulation stability. Because no single method establishes pore encapsulation, sorption and spectroscopic data should be integrated with mass balance and desorption measurements. These analyses define the formulation but do not establish its behavior in plants [22].

### 3.2. Stimulus-Responsive Release and Biological Performance

MOF cargo release can proceed through pore diffusion, competitive displacement, framework dissolution, ligand exchange, or surface-gate opening. Passive diffusion provides limited stress selectivity, whereas dissolution couples ABA release to simultaneous exposure to metal and linker components. Polymeric or supramolecular coatings can suppress baseline leakage and introduce pH-, redox-, enzyme-, temperature- or light-responsive gating. Yang et al. [18] demonstrated multistimulus phytohormone release from a MOF equipped with supramolecular nanovalves, establishing the feasibility of gated delivery but not drought-responsive signaling.

ABA@MIL-100(Fe) provides the most direct stress-related example. The reported ABA loading content was 22.2%, with 70.5% released within 3 h at pH 7.2, and the formulation improved drought-related traits in cotton seedlings [21]. However, release was measured by dialysis in an ethanol-containing phosphate buffer. Performance must therefore be reassessed in simulated apoplastic fluids spanning physiologically relevant pH, ionic strength and metabolite composition because organic acids, sugars, amino acids and competing ligands may alter ABA binding, framework dissolution and metal speciation.

MOF components may also affect plants independently of ABA. Empty MIL-100(Fe) promoted cotton seed germination [21], while metal–BTC frameworks produced node-dependent effects in fababean, with Cu-BTC promoting growth and Cr-BTC increasing oxidative-stress indicators [43]. Changes in biomass, chlorophyll or antioxidant activity therefore cannot be attributed to ABA delivery without appropriate controls. The minimum design should include drought-only, free-ABA, empty-MOF, metal-salt, free-linker and physical ABA–MOF-mixture treatments. A compositionally matched but non-responsive carrier would further distinguish triggered release from carrier chemistry.

Biological performance requires synchronization of drought development, ABA release, cellular uptake and signaling, and ABA removal during recovery. An effective carrier should exhibit low baseline leakage, rapid release in response to a validated drought cue, and minimal residual release after rewatering. Formulations should therefore be evaluated by trigger threshold, release lag, peak and residual release, target-tissue ABA exposure, SnRK2 activation and post-stress recovery rather than loading capacity alone. Table 1 distinguishes direct ABA-delivery evidence from supporting carrier studies and identifies the remaining mechanistic gaps. 

## 4. Drought-Responsive Release Triggers

### 4.1. Apoplastic pH and Redox Signals

Water deficit can alter xylem and apoplastic pH by affecting proton transport, ionic balance, nutrient uptake, and transpiration. Alkalinization increases the proportion of ABA^−^, promoting apoplastic retention, reducing passive membrane diffusion, and increasing reliance on ABA transporters for intracellular receptor access. In MOF systems, pH may regulate release by altering host–guest interactions, coordination bonds, or framework dissolution, although the direction and magnitude of this response are framework-specific. ABA release from MIL-100(Fe) was faster at pH 7.2 than at pH 4.5, but this response was measured in vitro using an ethanol-containing phosphate buffer [21]. Its biological relevance therefore depends on whether the corresponding pH range occurs at the intended delivery site. Because apoplastic and xylem pH vary among crops, organs, developmental stages, and drought intensities, trigger thresholds should be established using spatially resolved in-plant measurements and validated in physiologically realistic apoplastic fluids. Apoplastic alkalinization should thus be treated as a dynamic, context-dependent signal rather than a universal drought marker [50].

Drought and ABA signaling also modify reactive oxygen species dynamics, particularly hydrogen peroxide production in guard cells and the apoplast. Hydrogen peroxide promotes Ca^2+^ influx and ion-channel regulation during ABA-induced stomatal closure, providing a potential trigger for redox-responsive release [51]. Oxidation-sensitive boronate or thioketal gates may be suitable for this purpose, whereas disulfide gates respond primarily to reducing thiols and may be poorly matched to the apoplastic environment. Importantly, bulk-leaf hydrogen peroxide does not represent the redox conditions at the carrier surface. Fe- and Cu-containing MOFs may also catalyze Fenton-type or peroxidase-like reactions, modifying the signal they are intended to detect. Redox-gated systems must therefore quantify local hydrogen peroxide, ABA release and oxidative injury using empty-carrier controls to confirm responsiveness within the physiological redox range.

### 4.2. Ionic, Hydration, and Multistimulus Responses

Water deficit alters apoplastic solute composition, membrane potential, Ca^2+^ dynamics and ion fluxes. Competing anions, including phosphate, carbonate and organic acids, can destabilize susceptible MOFs through ligand exchange, protonation or metal sequestration. Such transformation may enable responsive release, but uncontrolled dissolution in application water, xylem sap or apoplastic fluid could liberate ABA before drought develops. Ionic responsiveness must therefore be engineered deliberately and evaluated through concurrent mass balances of the parent framework, ABA, metal nodes and linkers [22]. Hydration is a less specific trigger because particle water content is influenced by spraying, rainfall, dew and atmospheric humidity as well as plant water status. Hydrophilic coatings may swell and release ABA during application or rewatering, whereas dehydration-induced cracking may enhance release during drying but can be irreversible. Hydration-responsive designs are therefore biologically meaningful only when particle hydration and release are quantitatively related to target-tissue water potential under realistic environmental conditions.

Enzyme-sensitive MOFs demonstrate that biological gating is feasible. For example, cellulase-responsive coatings have been used for pathogen-targeted pesticide delivery [48], although infection-associated enzymes cannot be assumed to indicate drought. Multistimulus gating could improve specificity by requiring two concurrent signals, such as apoplastic alkalinization and elevated hydrogen peroxide. However, this approach increases synthetic complexity, batch variability, and validation requirements. Near-term designs should therefore prioritize one physiologically validated drought trigger combined with a coating that improves adhesion and suppresses premature leakage. It is important to distinguish between direct intracellular sensing by intact MOF particles and extracellular or apoplastic stimulus-responsive release. Currently, there is limited evidence demonstrating that intact MOF carriers enter plant cells and directly detect intracellular drought-associated signals. Therefore, MOF–ABA systems should be considered primarily as carriers that respond to drought-induced changes in their surrounding microenvironment, particularly at the leaf surface, apoplast, or other extracellular compartments. Drought-associated alterations, including changes in apoplastic pH, redox status, ionic composition, and hydration state, represent important components of plant stress signaling and may influence carrier behavior at the plant interface [50,52]. In responsive MOF systems, ABA release is unlikely to occur simply through weakened ABA–carrier interactions or passive enlargement of pore structures. Instead, drought-associated environmental changes may regulate ABA liberation through multiple physicochemical processes, including modification of metal–ligand coordination, changes in host–guest interactions, competitive displacement of guest molecules, framework flexibility, partial structural transformation, diffusion, or opening of responsive surface gates [41,53,54]. Following rehydration, restoration of the local microenvironment may reduce these activation signals, limiting further carrier transformation and attenuating ABA release. However, whether such reversible stimulus–response behavior can be achieved in plant systems remains to be experimentally demonstrated. Future validation should integrate measurements of drought-associated signals, MOF structural changes, ABA release kinetics, carrier transformation, and plant ABA responses during drought–rewatering cycles.

### 4.3. Biologically Realistic Release Assays

**Figure 2:**
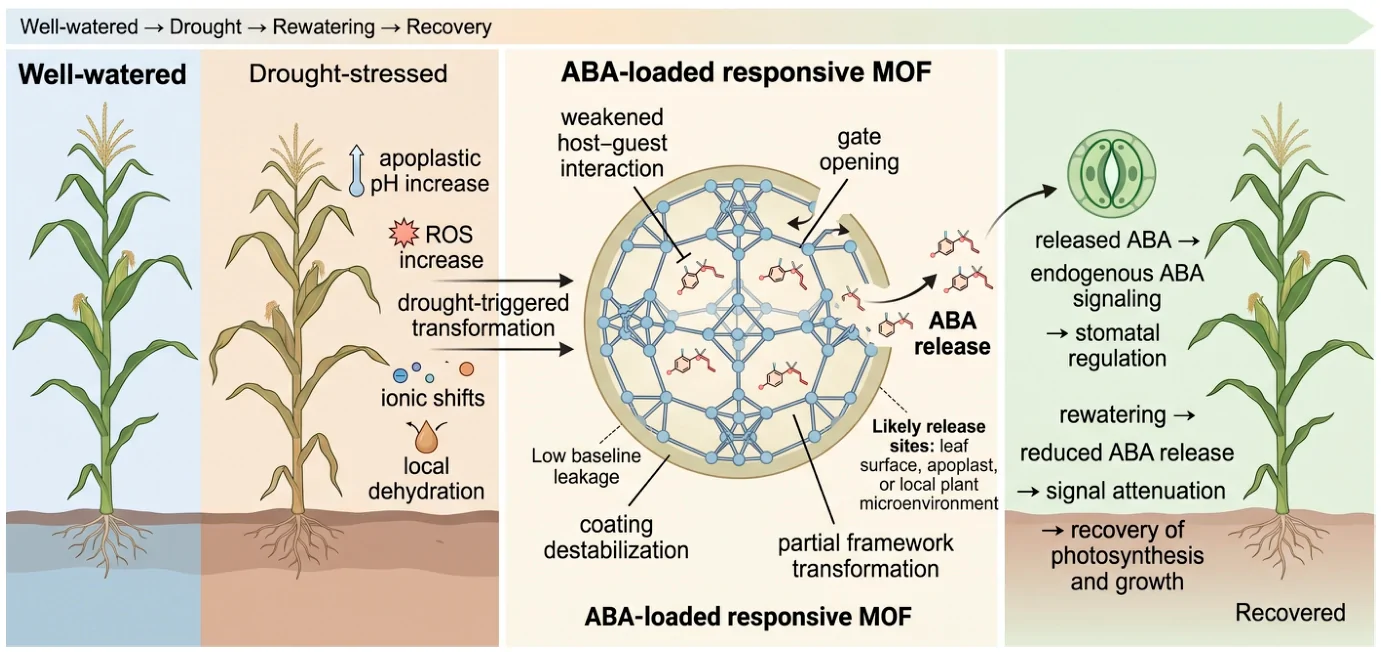
Drought-responsive ABA release from MOF carriers. The schematic illustrates how drought-associated cues act on an ABA-loaded MOF, the carrier-level changes that facilitate ABA release, and the subsequent decline in release following rewatering.

Release kinetics measured in distilled water or simple buffers cannot reliably predict MOF behavior at plant interfaces. Validation should progress from application water and simulated leaf-surface solutions to synthetic or collected apoplastic fluid or xylem sap, excised tissues, and intact plants. Light, temperature, relative humidity, spray deposition, droplet drying, and the ionic composition of the surrounding medium should reproduce the intended application route because these factors govern formulation persistence, foliar penetration, and transport across plant barriers [55,56,57,58,59]. Kinetic models can support formulation comparisons but cannot replace component-specific mass balance or biological validation. Released ABA must be linked to target-tissue exposure, PYR/PYL/RCAR–PP2C–SnRK2 activation, stomatal dynamics, and recovery after rewatering. A biologically effective profile should generate the intended ABA pulse without persistent signaling or carrier-induced injury. Together, these considerations indicate that drought-responsive ABA delivery should be viewed as a sequence linking a physiologically relevant stress cue to a defined physicochemical response of the carrier and subsequent ABA release. Responsive release need not require intracellular sensing by intact MOF particles; it may instead occur at the leaf surface, within the apoplast, or in other local plant microenvironments where drought-associated changes in pH, redox state, ionic composition, or hydration can alter host–guest interactions, responsive gates or coatings, and framework stability. Figure 2 summarizes these proposed trigger-to-release mechanisms and illustrates how their attenuation after rewatering could reduce ABA release and facilitate downstream physiological recovery.

## 5. Coupling ABA Release to PYR/PYL/RCAR-PP2C-SnRK2 Signaling

### 5.1. ABA Perception and Context-Dependent SnRK2 Activation

ABA binds the conserved hydrophobic pocket of PYR/PYL/RCAR receptors, inducing closure of the gate and latch loops and enabling interaction with clade A PP2Cs [60,61,62]. The ABA–receptor complex inhibits the PP2C catalytic site, releasing downstream SnRK2 kinases from phosphatase-mediated repression [63,64]. For MOF delivery, the relevant variable is therefore receptor-accessible ABA within target cells rather than total tissue ABA. Because receptor abundance, affinity and PP2C composition vary among tissues, equivalent whole-leaf ABA concentrations may produce different receptor occupancy and signaling outputs. ABA measurements should therefore be integrated with genetically encoded sensors, receptor-dependent reporters, receptor mutants or downstream phosphorylation assays [60,63,65].

At low ABA concentrations, clade A PP2Cs maintain subclass III SnRK2s in a dephosphorylated state. ABA-bound receptors inhibit PP2Cs, permitting activation of SnRK2.2, SnRK2.3 and SnRK2.6/OST1. Simultaneous loss of these kinases causes profound ABA insensitivity and defects in drought response, development and reproduction [37,38]. SnRK2 activation also requires RAF-like MAPKKK inputs. B2/B3 RAFs preferentially activate ABA-responsive subclass III SnRK2s, whereas B4 RAFs primarily activate ABA-unresponsive subclass I SnRK2s during osmotic stress [27]. Functionally redundant B3 MAPKKKs are also required for robust activation of subclass III SnRK2s by ABA and osmotic stress [28]. The SnRK2 activation threshold therefore depends on ABA exposure, PP2C repression, RAF activity, and osmotic context rather than on ABA concentration alone [66,67]. SnRK2 abundance must also be distinguished from kinase activity. Transcript and total-protein measurements cannot establish pathway activation, which depends on phosphorylation state, protein interactions, and catalytic function. Claims that an ABA-loaded MOF activates SnRK2 signaling should therefore require phosphosite-specific or Phos-tag immunoblotting, kinase assays, phosphoproteomics, validated reporters, or genetic dependence rather than qPCR alone [27,68]. The strongest evidence would quantitatively relate target-cell ABA exposure to the onset, amplitude, and duration of SnRK2 phosphorylation.

### 5.2. Guard-Cell, Transcriptional, and Hydraulic Responses

OST1/SnRK2.6 is a major ABA-activated kinase in guard cells that phosphorylates the S-type anion channel SLAC1 [69,70]. Anion efflux depolarizes the plasma membrane, activates outward K^+^ transport, and drives osmotic water efflux, reducing guard-cell turgor and closing stomata. Structural analysis has further defined the pore architecture underlying SLAC1 selectivity and gating [71]. ABA also stimulates ROS production through RBOHD and RBOHF, reinforcing Ca^2+^ influx and ion-channel regulation [72]. Carrier-induced ROS must therefore be interpreted spatially and temporally: a transient guard-cell ROS pulse may support stomatal closure, whereas persistent accumulation may indicate oxidative injury. Stomatal aperture, conductance, leaf temperature, transpiration, and CO_2_ assimilation should be monitored throughout the drought and rewatering periods. Rapid closure may preserve water status, but delayed reopening restricts cumulative carbon gain. The desired response is therefore timely closure followed by rapid recovery rather than maximal or sustained stomatal limitation [69,70].

Activated SnRK2s also phosphorylate AREB/ABF transcription factors and, in seeds, ABI5, which regulate ABA-responsive genes involved in dehydration protection, osmotic adjustment and pathway feedback. A transient kinase pulse may generate transcriptional responses that persist after ABA declines, whereas continuous release may prolong growth inhibition. Resolving this temporal relationship requires integrated measurements of ABA, phosphorylation and gene expression. Omics profiles alone cannot establish carrier-specific causality and should be supported by time-matched comparisons of free ABA, empty MOF, physical mixtures, non-responsive formulations and responsive ABA-loaded MOFs. ABA also regulates plant hydraulics through tissue-specific effects on aquaporin abundance, trafficking and phosphorylation, root water uptake and xylem function [73]. Root-derived, vascularly transported and leaf-synthesized ABA collectively shape long-distance drought signaling [9,50]. Foliar delivery is suited to rapid guard-cell regulation, whereas root delivery may influence xylem transport and root hydraulics but is more susceptible to sorption and rhizosphere transformation. Claims of whole-plant hydraulic protection should therefore include leaf and root hydraulic conductance, water potential, relative water content and soil water depletion rather than antioxidant markers alone.

### 5.3. Photosynthetic Protection and Post-Drought Recovery

ABA-induced stomatal closure limits water loss and can preserve leaf water potential and photosystem function, but it also restricts CO_2_ diffusion and carbon assimilation. This trade-off depends on stress intensity and duration: hydraulic protection may dominate during severe drought, whereas sustained closure under mild stress or recovery reduces growth. MOF-mediated ABA delivery should therefore be evaluated using integrated measurements of gas exchange, chlorophyll fluorescence, leaf water status, biomass and yield. Increased instantaneous WUE caused by near-complete stomatal closure should not be interpreted independently as an agronomic benefit. Recovery provides the most stringent test of programmed delivery. As endogenous ABA declines after rewatering, PP2C activity is restored, SnRK2 phosphorylation decreases, and stomata reopen. Continued ABA release from an external reservoir may maintain pathway activation and delay photosynthetic recovery. A responsive MOF should therefore raise ABA above the receptor–SnRK2 activation threshold during drought and rapidly reduce exposure below this threshold after rewatering. Time-resolved measurements of ABA release, target-tissue ABA, SnRK2 phosphorylation, stomatal conductance and photosynthesis across repeated drought–rewatering cycles are required to test this behavior. The defining performance criterion is rapid protective activation without prolonged stomatal limitation during recovery.

## 6. Uptake, Transport, Transformation, and Fate

### 6.1. Foliar Retention, Entry and Root-Interface Transformations

For foliar delivery, MOFs must remain on the treated surface long enough to release ABA; intact-particle entry is required only when carrier translocation is intended. Leaf retention depends on wax composition, surface roughness, trichomes, stomatal distribution and environmental conditions, while particle size, morphology, charge and surface chemistry regulate droplet spreading, aggregation, adhesion and rainfastness. Biodegradable coatings can improve retention but may also alter ABA release and cuticular interactions [74]. Nanoparticle studies similarly demonstrate that particle size and coating chemistry independently affect foliar adhesion, entry and translocation [75].

Potential entry routes include the cuticle, stomata, hydathodes, trichome bases and damaged tissues, although their contributions vary among crops and application conditions. Nominal size-exclusion limits cannot be applied directly because MOFs may aggregate, fragment, or partially dissolve before entry. Detection of framework-derived metals in washed tissues therefore does not demonstrate uptake of intact particles. Rigorous washing, quantitative analysis of surface-recovery fractions, complementary imaging, and chemical speciation are required to distinguish surface-retained MOFs from dissolved metal ions and intact or transformed material internalized within plant tissues [76,77,78,79].

Root-applied MOFs encounter soil minerals, organic matter, microorganisms, root exudates, phosphate and pH gradients that can induce adsorption, aggregation, ligand exchange, dissolution or corona formation. Cell walls and the endodermal casparian strip restrict radial transport, although root tips, lateral-root emergence zones and damaged tissues may provide alternative entry routes. Intact MOFs, dissolved metals, linker fragments and released ABA may therefore follow distinct pathways. Root-delivery studies must quantify rhizosphere transformation and ABA release before interpreting tissue metal accumulation as carrier uptake.

### 6.2. Long-Distance Transport and Tissue-Resolved Delivery

After crossing external barriers, intact MOFs or their transformation products may move through apoplastic or symplastic pathways and, if vascular tissues are reached, through the xylem or phloem. Xylem transport is largely driven by transpiration, whereas phloem redistribution depends on vascular loading and source–sink relations. These pathways cannot be inferred from the application route. A foliar ABA-loaded MOF may function without systemic particle transport if ABA is released locally and subsequently enters endogenous transport pathways. By contrast, claims of carrier-mediated targeting to roots, reproductive organs or developing seeds require direct evidence distinguishing intact MOFs from released ABA, dissolved metals and linker products.

No single analytical method can simultaneously resolve localization, framework integrity and ABA origin. Fluorescence microscopy may track a dissociated label, while conventional ICP-MS measures total metal without distinguishing particles, ions or newly formed phases. Single-particle ICP-MS can detect particulate metal but does not confirm preservation of the original framework. Reliable localization therefore requires orthogonal combinations of elemental imaging, electron microscopy, X-ray absorption spectroscopy, vibrational mapping and independently labeled nodes and linkers. Label stability must be verified in formulation media and simulated plant fluids. Stable-isotope-labeled ABA coupled with liquid chromatography–tandem mass spectrometry is additionally required to distinguish delivered ABA from endogenous hormone pools [80]. Tissue targeting should therefore be demonstrated through convergent chemical and spatial evidence rather than a single fluorescent or elemental signal.

### 6.3. Carrier Transformation and ABA Fate

MOFs deposited on or entering plants are unlikely to retain their initial physicochemical state. They may aggregate, lose crystallinity, exchange ligands, acquire biomolecular coronas, partially dissolve, or become sequestered in cell walls and intracellular compartments. Phosphate-rich fluids and acidic compartments can destabilize susceptible coordination networks, whereas highly stable frameworks may persist and create harvest-stage residue concerns. Transformation is specific to both the framework and exposure environment and cannot be predicted from stability in water; simple carrier fate should be evaluated throughout the application cycle, from formulation and deposition to plant uptake, transformation and harvest. Key endpoints include intact-particle fraction, aggregation, crystallinity, dissolution kinetics, metal speciation, linker transformation products, tissue retention and remobilization. In edible crops, particulate residues, metals and linker-derived products should be quantified separately in harvested organs. Short-term seedling performance is insufficient to establish long-term safety or residue behavior.

**Figure 3:**
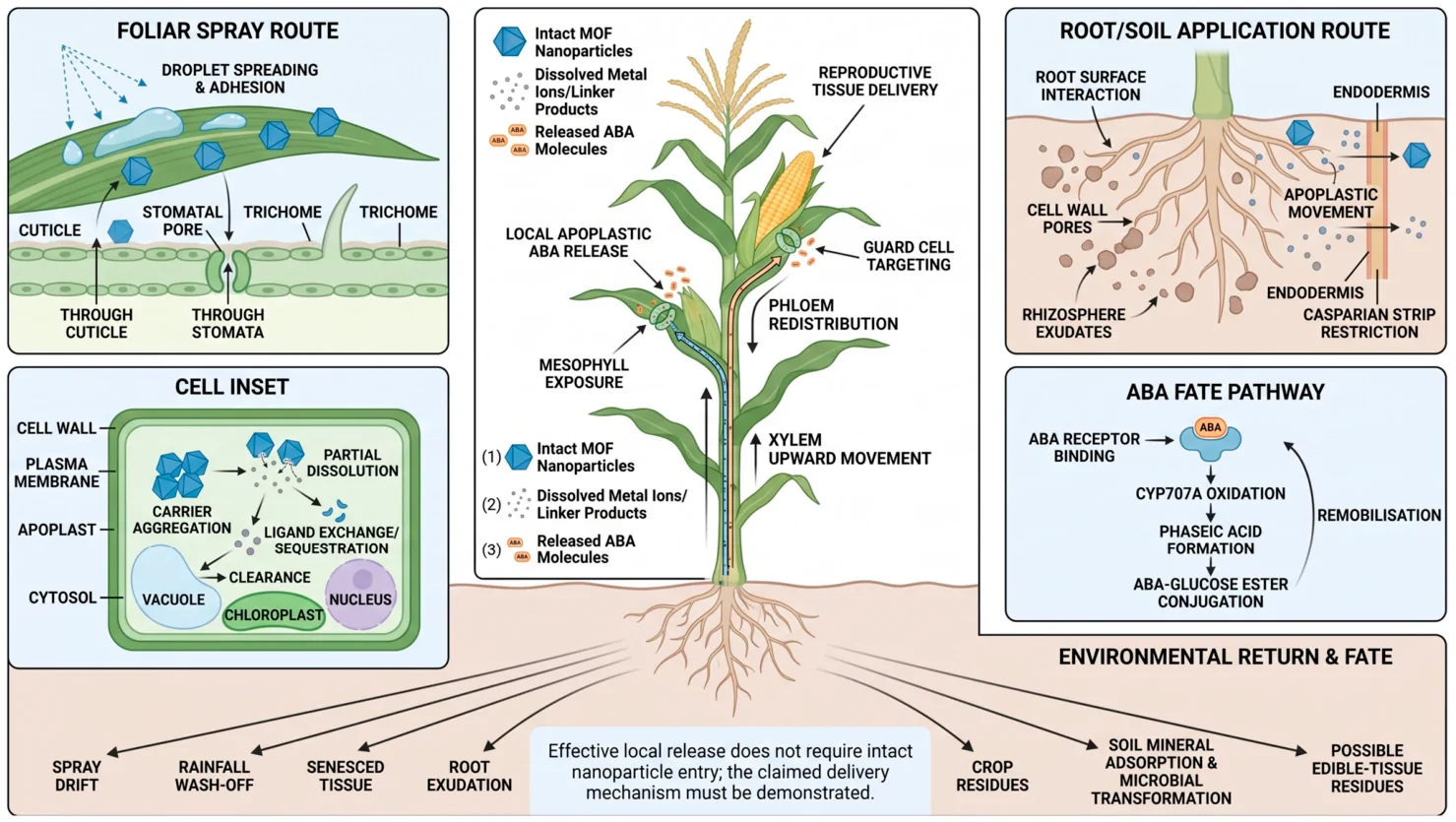
Uptake, transport, transformation, and fate of ABA-loaded MOFs in plants. Foliar and root application routes should be traced through surface retention, cuticular or stomatal entry, root apoplastic barriers, xylem and phloem transport, local ABA release, guard-cell and vascular signaling, framework dissolution, metal and linker fate, ABA metabolism, soil return, and possible edible-tissue residues. The figure should distinguish intact-particle movement, dissolved-ion movement, and locally released ABA.

Released ABA enters endogenous transport and metabolic pathways, where it may bind receptors, move between tissues, undergo CYP707A-mediated hydroxylation or become conjugated. Receptor-accessible ABA in guard cells or vascular tissues is therefore more informative than total leaf ABA. Stable-isotope tracing can distinguish delivered ABA and its metabolites from endogenous hormone pools and determine whether the formulation directly supplements active ABA, modifies its metabolism or induces endogenous synthesis. This distinction is essential because an empty MOF may trigger osmotic, ionic or oxidative stress and indirectly increase endogenous ABA. Isotope-resolved analyses should therefore be combined with empty-MOF, free-ABA, metal-salt, free-linker and non-responsive-carrier controls. Figure 3 distinguishes three fate pathways that require independent evaluation: intact carrier movement, transport of dissolved framework components and redistribution of locally released ABA.

## 7. Conserved ABA Signaling and Crop-Specific Formulation

### 7.1. Cereal Crops

SnRK2s are plant-specific serine/threonine kinases conserved across land plants and major crops [25]. The PYR/PYL/RCAR–PP2C–SnRK2 signaling architecture is broadly conserved, but its quantitative output varies through gene duplication, polyploidy, tissue-specific expression, and developmental regulation. Subclass III SnRK2s are generally strongly ABA-responsive, whereas subclass I and II members exhibit predominantly osmotic or mixed ABA sensitivity. This conservation supports a common mechanistic framework but not universal ABA thresholds or formulations. In rice, SnRK2 proteins are termed stress-activated protein kinases. OsSAPK2 regulates ABA sensitivity, stomatal behavior and drought tolerance, with loss-of-function mutants exhibiting greater water loss and stress susceptibility [81]. SAPK2 also influences nutrient metabolism, grain development and yield under reproductive-stage drought, linking kinase signaling to agronomic performance [35]. However, differences between flooded and upland production systems affect root-zone exposure, drought progression and carrier transformation, limiting direct extrapolation to other cereals.

Maize contains an expanded SnRK2 family in which *ZmSnRK2.10* participates in ABA signaling. Reciprocal regulation between *ZmCIPK33* and *ZmSnRK2.10* integrates Ca^2+^ signaling, PP2C regulation and *ZmSLAC1* activation during drought [82,83]. SnRK2 activation in maize must therefore be considered within a broader kinase network. Foliar formulations must also account for maize-specific cuticular properties, stomatal distribution and canopy development rather than relying solely on Arabidopsis guard-cell responses. Hexaploidy wheat contains numerous SnRK2 homeologs with distinct stress responsiveness. Subclass III TaSnRK2s preferentially interact with group A PP2Cs, confirming conservation of the canonical ABA module [84]. The TaPP2C01–TaSnRK2.4–TaABF2 and TaPP2C53-1A–TaSnRK2.8-5A–TabZIP23-6D modules further demonstrate homeolog-specific signaling functions [85,86]. Wheat formulations should therefore be matched to the dominant signaling module in the intended tissue and developmental stage.

### 7.2. Eudicot Crops

Cotton provides the most direct evidence for MOF-mediated ABA delivery under drought [21]. Other eudicot crops possess conserved ABA-responsive SnRK2 families but differ substantially in leaf surface properties, stomatal distribution, root biology and harvested organs. Legumes introduce nodulation and rhizosphere interactions, while leafy vegetables, fruits and oilseeds require distinct assessments of reproductive sensitivity and harvest-stage residues. Cross-crop translation must therefore distinguish pathway conservation from formulation equivalence. The PYR/PYL/RCAR–PP2C–SnRK2 module provides a shared mechanistic scaffold, but particle retention, ABA uptake, activation thresholds, recovery kinetics and acceptable residue levels must be established for each crop and application route. Table 2 summarizes crop-specific signaling evidence and formulation constraints.

## 8. Temporal Signaling Across Drought and Recovery

### 8.1. ABA Trajectories and Carbon Costs

Optimal ABA exposure is phase-dependent. Premature release can restrict stomatal conductance, carbon assimilation, and growth before stress develops. During early soil drying, a rapid ABA increase can reduce water loss, while continued signaling during progressive drought may sustain hydraulic protection and protective gene expression. After rewatering, receptor-accessible ABA should decline, PP2C activity should recover, SnRK2 phosphorylation should decrease, and stomata should reopen. Sustained release is therefore beneficial only while water deficit persists. Formulations must be evaluated across the complete drought–rewatering cycle. Free ABA typically produces rapid exposure followed by rapid loss, whereas passive slow-release systems can prolong signaling independently of plant water status. A stress-programmed carrier should instead exhibit low baseline leakage, trigger-dependent release and rapid attenuation after the trigger subsides. These profiles must be compared at equal ABA doses using peak concentration, time to peak, tissue ABA area under the curve, duration above the SnRK2 activation threshold, and residual exposure after rewatering. ABA exposure should be matched temporally with SnRK2 phosphorylation, stomatal conductance, and photosynthesis. Reciprocal regulation between ABA and TOR signaling provides a molecular basis for the transition from stress protection to renewed growth [87]. Instantaneous WUE alone cannot resolve this trade-off because strong stomatal limitation may increase the ratio while severely restricting carbon gain. Recovery should instead be evaluated through cumulative assimilation, restoration of pre-stress gas exchange, leaf expansion, biomass, reproductive development and yield. The optimal formulation should preserve hydraulic integrity while minimizing integrated carbon loss and post-stress growth delay.

### 8.2. Hormonal Crosstalk and Repeated Drought

**Figure 4:**
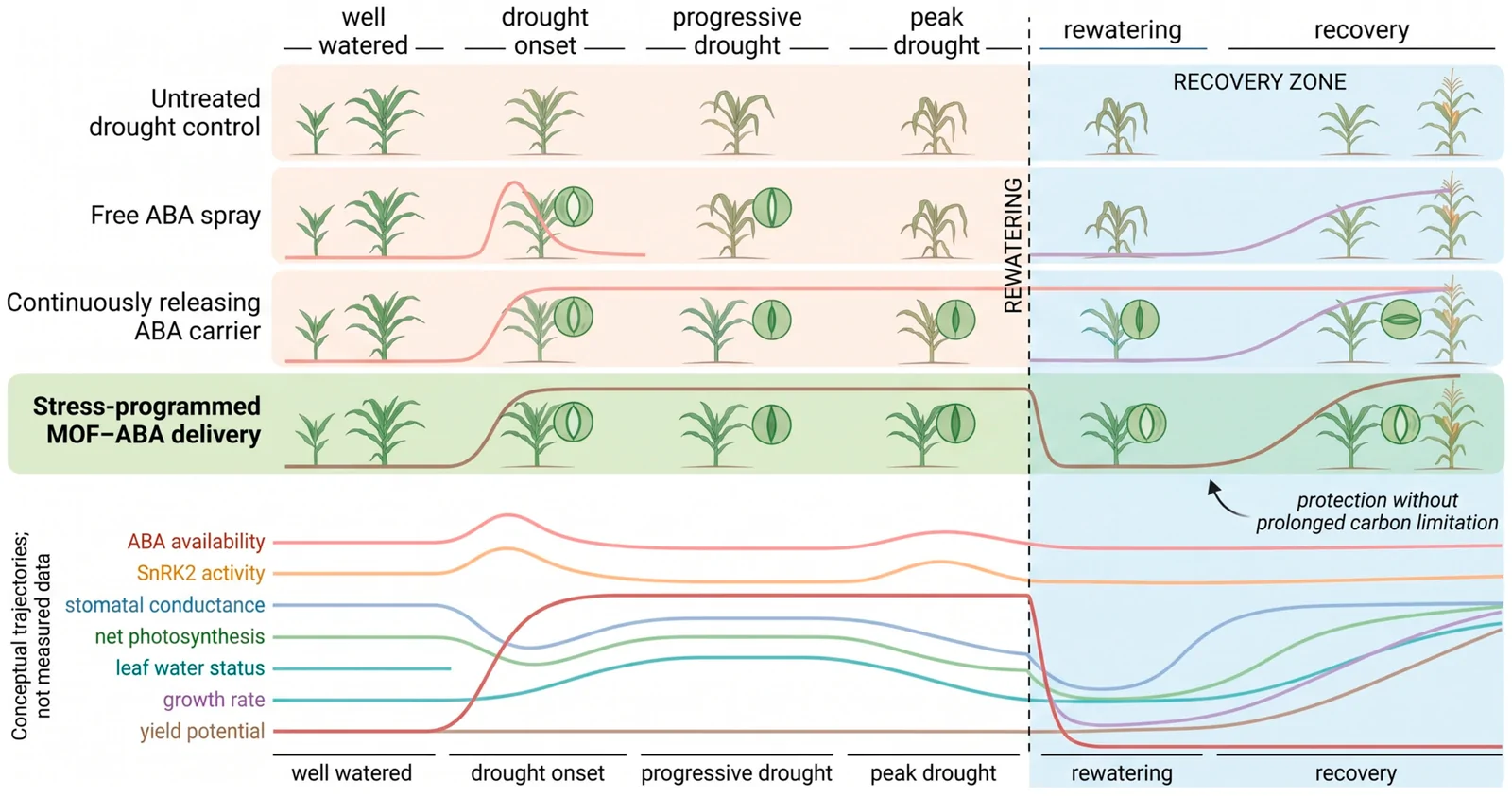
Temporal regulation of the drought survival–growth trade-off. Compare untreated drought, free ABA, a continuously releasing ABA carrier, and a stress-programmed MOF–ABA system across pre-stress, drought onset, progressive drought, peak drought, rewatering, and recovery. Conceptual trajectories should show ABA availability, SnRK2 activation, stomatal conductance, photosynthesis, plant water status, growth, and yield potential. The desired design produces a protective ABA pulse during drought and rapid signal attenuation after rewatering.

ABA interacts with auxin, gibberellins, brassinosteroids, jasmonates and ethylene to regulate root development, senescence, reproduction and growth recovery. Sequential delivery of ABA during drought and a growth-promoting hormone during recovery is conceptually possible but remains speculative. Multihormone systems should be considered only after the release kinetics, signaling effects and recovery profile of an ABA-only formulation have been established. Crops frequently experience repeated drying and rewatering. Previous drought can modify guard-cell behavior and transcriptional responses, creating physiological memory that alters subsequent stress responses [88]. Persistent carriers may also accumulate or remain active between episodes. Repeated-cycle experiments should therefore compare untreated plants, free ABA, empty MOFs, passive-release formulations and stress-responsive ABA-loaded MOFs. Longitudinal measurements should include carrier persistence, ABA exposure, SnRK2 phosphorylation, stomatal conductance, photosynthetic recovery, growth and metal or linker accumulation. A persistent carrier is advantageous only if it responds selectively to each drought episode and returns to a low-release state after rewatering. Figure 4 integrates material residence, ABA exposure, signaling and physiological recovery across repeated cycles.

## 9. Evidence Boundaries and Claim-Matched Validation

### 9.1. Material Feasibility Versus Pathway-Level Evidence

Direct evidence for MOF-mediated phytohormone delivery remains limited. Multistimulus MOFs have enabled gated gibberellin release, while ABA@MIL-100(Fe) improved drought-related performance in cotton seedlings [18,21]. Coordination polymers and AgroMOFs incorporating plant-active ligands, including 3-indolepropionic acid and L-mimosine, provide additional evidence of material feasibility [44,45]. These studies do not, however, establish precise control of phytohormone signaling across crops.

The cotton study demonstrated ABA loading, UV photoprotection under controlled conditions, pH-dependent release and physiological effects, but did not resolve exogenous versus endogenous ABA, carrier localization, receptor engagement, SnRK2 activation or reproductive yield. Physiological endpoints such as biomass, relative water content, antioxidant activity and survival are important but mechanistically nonspecific because they may reflect ABA activity, metal nutrition or carrier-induced stress. Likewise, SnRK2 transcription does not demonstrate kinase activation. Claims of PYR/PYL/RCAR–PP2C–SnRK2 engagement should require evidence of receptor dependence, PP2C inhibition, SnRK2 phosphorylation or activity, downstream substrate phosphorylation, or genetic loss of response.

### 9.2. Uptake Evidence and Essential Controls

Detection of framework-associated metals or ABA in whole tissues does not prove intact MOF uptake. Surface retention, framework dissolution and ion transport can produce similar signals. Carrier localization and transformation therefore require convergent microscopic, elemental and spectroscopic evidence, while fluorescence imaging is reliable only when dye retention, photostability and carrier–label integrity have been demonstrated under the relevant biological conditions [77,78,89,90]. The minimum experimental design should include drought-only, free-ABA, empty-MOF, ABA-loaded-MOF, physical ABA–MOF-mixture, metal-salt, and free-linker treatments. A compositionally matched non-responsive or altered-trigger carrier should be included where possible to isolate the contribution of stimulus-dependent release. ABA dose and framework-component exposure must be matched across treatments. The drought system must also be defined precisely because osmotic assays, detached tissues, hydroponics, soil drying and field drought impose distinct physiological constraints and cannot support equivalent conclusions.

### 9.3. Minimum Validation Standards

Validation should be proportional to the claim. Formulation-level evidence requires reproducible synthesis, loading mass balance, ABA integrity, release in biologically realistic media and quantification of metal and linker release. Delivery-level evidence should establish surface retention, local ABA release or carrier internalization using spatial and source-resolved measurements. Mechanistic evidence must connect ABA exposure to receptor–PP2C–SnRK2 activation rather than relying solely on transcriptional or antioxidant responses. Physiological studies should integrate water relations, gas exchange and post-stress recovery, while agronomic studies should assess reproductive development and yield. Safety evaluation should address edible-tissue residues, soil transformation, non-target organisms and microbiome effects. Narrower studies remain valuable when their conclusions are restricted to the evidence generated. Table 3 summarizes these requirements as a graded validation framework.

## 10. Biosafety, Environmental Fate, and Field Translation

### 10.1. Phytotoxicity, Residues, and Environmental Transformation

MOF biocompatibility is determined by dose, exposure duration, dissolution kinetics, and transformation rather than by the nutritional status of the metal node. Fe, Zn, and Cu may become phytotoxic when released rapidly or accumulated locally, while linkers and coatings can alter membrane integrity, redox homeostasis, and microbial activity. Empty-carrier dose–response studies should therefore assess photosynthesis, membrane damage, ion balance, growth and reproduction rather than visible injury alone. Carrier stability presents a further trade-off: rapid degradation can generate high local concentrations of ABA, metals and linkers, whereas persistent particles may accumulate. Degradation kinetics must therefore support delivery while maintaining all components below adverse-effect thresholds.

Because treated crops enter food and feed chains, residue analysis should quantify ABA and its metabolites, total and speciated metals, linker-derived products and, where possible, intact particles [80,92]. Surface residues are particularly important in leafy vegetables, whereas cereals, oilseeds and fruit crops require assessment of translocation into harvested organs. Foliar-applied MOFs can reach soil through drift, wash-off and crop residues, while root-applied formulations interact directly with soil minerals, organic matter and microorganisms. In soil, MOFs may dissolve, adsorb to mineral surfaces or form new chemical phases. Environmental assessment should therefore quantify metal and linker persistence, repeated accumulation, soil enzyme activity, nutrient cycling and effects on rhizosphere and phyllosphere microbiomes [93].

### 10.2. Field Performance, Regulation and Responsible Design

Field performance depends on ultraviolet exposure, rainfall, temperature, humidity, water chemistry, leaf dust, canopy structure and application equipment. Alkaline or hard water may trigger premature release, destabilize frameworks or promote aggregation, while tank mixtures introduce competing ions, ligands and surfactants. Formulations should be evaluated for shelf life, suspension stability, nozzle compatibility, leaf coverage, rainfastness and efficacy under fluctuating field conditions. Production costs and energy requirements for synthesis, purification, activation and coating must also be compatible with agricultural use [92]. Regulatory classification may vary depending on whether the formulation is considered a plant growth regulator, nanomaterial, fertilizer or pesticide. Standardized terminology, material characterization, exposure measurements and transformation data will therefore be required. Formulation design should prioritize abundant, low-risk metals, biodegradable or biologically derived ligands, aqueous synthesis, minimal effective doses, degradable coatings and life-cycle assessment [92]. These criteria should be integrated during development rather than evaluated only after efficacy has been established.

## 11. Conclusions and Research Priorities

### 11.1. Conclusions

Stimulus-responsive MOFs provide a promising strategy for coupling endogenous drought-associated signals with dynamically regulated ABA delivery. However, their biological value should not be evaluated solely based on ABA loading capacity or prolonged release. The desired outcome is a spatially and temporally controlled ABA pulse that is activated during drought and attenuated after rewatering to support recovery while avoiding prolonged stomatal limitation and reduced carbon assimilation.

MOF-mediated ABA delivery should therefore be considered within the broader drought signaling network, where changes in apoplastic pH, ROS and redox status, Ca^2+^ dynamics, ionic composition, and hydration state influence ABA perception and downstream responses. Importantly, current advances in MOF-based ABA delivery have established the feasibility of controlled hormone protection and release, while mechanistic integration of drought-associated cues, carrier activation, ABA availability, and PYR/PYL/RCAR–PP2C–SnRK2 pathway activation remains an emerging research direction. Current evidence remains limited to ABA-loaded MOF formulation, controlled release characteristics, and associated physiological improvements.

Future studies should move beyond carrier performance assessment and establish mechanistic connections between MOF transformation, ABA delivery, pathway activation, physiological recovery, yield performance, and environmental safety. Such integrated validation will determine whether responsive MOFs can function as biologically informed interfaces between drought perception and hormone signaling for improving crop resilience under variable water availability.

### 11.2. Research Priorities

1. Define physiologically relevant drought triggers. Apoplastic pH, ROS and redox status, Ca2+ and ionic dynamics, and tissue hydration during drought development and rewatering should be spatially and temporally resolved. Responsive release should be tested within the actual physiological ranges encountered in target tissues rather than under arbitrary buffer conditions, with trigger thresholds, response lag, baseline leakage, release amplitude, and reversibility quantified.
2. Synchronize material behavior with ABA signaling dynamics. MOF stability and ABA-release kinetics should be aligned with drought progression, hormone uptake, receptor accessibility, SnRK2 activation, and ABA removal during recovery. Responsive systems should be compared with free ABA, empty carriers, and passive-release formulations at equivalent ABA doses to determine whether stress-responsive delivery provides a genuine temporal advantage.
3. Resolve the origin, localization, and fate of delivered ABA. Stable-isotope-labeled ABA should be combined with tissue-resolved imaging, chemical speciation, and quantitative mass balance to distinguish exogenously delivered ABA from endogenous synthesis, conjugated pools, and catabolic products. Parallel analyses should differentiate intact carrier movement from framework dissolution, released metal ions, linker products, and locally released hormone.
4. Establish molecular causality in the ABA–SnRK2 pathway. Evidence of pathway engagement should extend beyond transcript abundance. Receptor dependence, PP2C inhibition, SnRK2 phosphorylation or kinase activity, and phosphorylation of downstream targets such as ion channels and AREB/ABF transcription factors should be quantified. Genetic, pharmacological, or reporter-based approaches should be used wherever possible to confirm that physiological effects depend on the intended signaling pathway.
5. Connect signaling with whole-plant physiology and recovery. Time-resolved measurements should integrate ABA exposure and SnRK2 activity with ROS–Ca2+ dynamics, stomatal conductance, transpiration, photosynthesis, chlorophyll fluorescence, plant water status, hydraulic function, antioxidant regulation, and stress-responsive transcription. Particular attention should be given to stomatal reopening and restoration of carbon assimilation after rewatering because prolonged protection at the expense of recovery would limit agronomic value.
6. Develop crop- and stage-specific formulations. Responsive delivery should account for differences in leaf anatomy, cuticle composition, stomatal distribution, root architecture, ABA sensitivity, SnRK2 family composition, developmental stage, and reproductive vulnerability. Horticultural crops, cereals, legumes, and other crop groups may therefore require distinct trigger thresholds, application routes, doses, and recovery profiles rather than a universal MOF–ABA formulation.
7. Validate agronomic resilience and sustainable field translation. Performance should be evaluated across repeated drought–rewatering cycles and under realistic variation in radiation, temperature, rainfall, humidity, spray-water chemistry, and soil conditions. Studies should extend beyond seedling survival to reproductive development, yield stability, water productivity, and product quality. Environmental assessment should simultaneously address edible-tissue residues, metal and linker transformation, soil and microbiome effects, repeated accumulation, production cost, biodegradability, and life-cycle impacts.

Collectively, these priorities can move the field from proof-of-concept hormone delivery toward mechanistically validated, crop-specific, and environmentally responsible systems that connect drought sensing with dynamic ABA signaling and sustainable crop resilience.

## Figures and Tables

**Table 1:** Direct and supporting evidence for MOF-mediated delivery of plant hormones and growth regulators.

System	Plant/Objective	Loading and Trigger	Results	Mechanistic Strength and Main Limitation	Reference
ABA@MIL-100(Fe)	Cotton; drought mitigation	ABA loading 22.2%; faster release at pH 7.2; ultraviolet protection	Improved seedling drought performance after foliar application	Direct ABA–MOF evidence, but no source-resolved ABA, receptor assays, SnRK2 phosphorylation, recovery, yield, or residue analysis	[21]
GA-loaded supramolecular MOF nanoplatform	Wheat and Chinese cabbage; growth regulation	Macrocycle nanovalves; pH, competitive binding, and light-related stimuli	Promoted plant growth and demonstrated multistimulus regulator delivery	Direct plant-regulator delivery; not designed around drought physiology or ABA–SnRK2 signaling	[18]
Cu- and Zn-indole propionate coordination polymers	Model agricultural systems; root-growth regulator delivery	Plant-active ligand incorporated into coordination structure	Demonstrated a route for integrating a growth regulator with micronutrient metals	Direct coordination-polymer evidence; plant uptake, stress-specific release, and receptor-level action remain limited	[44]
Zn-mimosinate AgroMOF	Controlled agrochemical delivery	L-mimosine functions as the organic component; stable Zn-based framework	Provides a multifunctional plant-active ligand and micronutrient carrier concept	Strong material evidence; crop stress mechanism and environmental fate require direct validation	[45]
Fe-based porous MOF fungicide carrier	Crop protection and micronutrient supply	Controlled fungicide release from Fe-containing porous framework	Combined disease control with crop nutritional function	Supports multifunctional carriers, but active-ingredient action and metal nutrition are confounded	[46]
Protease-triggered MOF hybrid	Long-term pest management and micronutrient supply	Protease-responsive release	Extended pest control with micronutrient co-delivery	Demonstrates enzyme-responsive agricultural release; trigger is pathogen-related rather than drought-specific	[47]
Clothianidin-loaded ZIF-8	Long-term insect control	One-pot encapsulation; pH-sensitive ZIF-8 matrix	Improved persistence and biological efficacy	Relevant carrier chemistry, but not hormone delivery or drought signaling	[48]
Functionalized Zn-MOF with ortho-disulfides	Controlled agricultural delivery	One-step encapsulation in functionalized Zn framework	Demonstrated MOF-enabled delivery of bioactive molecules	Useful design precedent; crop uptake, transformation, and non-target safety need stronger evidence	[49]

**Table 2:** Conservation and functional variation in ABA–SnRK2 signaling across representative monocot and eudicot crops.

Species/Crop	Representative ABA-Responsive SnRK2 Module	Main Demonstrated Function	Evidence Type	Implication for MOF–ABA Delivery	Reference
*Arabidopsis thaliana*	PYR/PYL–PP2C–SnRK2.2/2.3/2.6; OST1/SnRK2.6–SLAC1	ABA perception, kinase activation, stomatal closure, transcription, and growth–stress balance	Receptor structures, mutants, biochemistry, phosphoproteomics, and electrophysiology	Best system for causal validation; crop translation still requires delivery and anatomical adjustment	[37,60,63,71]
Rice	*OsSAPK2* and related SAPKs	ABA sensitivity, stomatal regulation, osmolyte accumulation, redox protection, reproductive drought performance	Knockout, overexpression, physiology, and yield-related analyses	Provides a cereal bridge from kinase mechanism to agronomic outcome	[35,81]
Maize	ZmSnRK2.10–ZmCIPK33–ZmPP2C11–ZmSLAC1	ABA signaling, Ca^2+^ crosstalk, anion-channel regulation, and drought resistance	Genome-wide family analysis, protein interaction, kinase assays, and genetics	High-value translational crop; amphistomatous leaves and reproductive drought require formulation-specific tests	[82,83]
Wheat	TaPP2C01–TaSnRK2.4–TaABF2; TaPP2C53-1A–TaSnRK2.8-5A–TabZIP23-6D	Stomatal regulation, osmotic adjustment, redox homeostasis, root traits, biomass, and yield association	Protein interactions, transgenic analysis, field panels, and gene regulation	Polyploid redundancy requires member-specific biomarkers and careful dose–response analysis	[84,85,86]
Cotton	Conserved ABA–PP2C–SnRK2 network; direct ABA@MIL-100(Fe) evidence	Seedling drought protection and carrier-derived Fe effects	Direct formulation bioassay plus established ABA physiology	Most direct proof of concept; still lacks kinase-level causality, recovery, yield, and residue data	[21]
Soybean and legumes	Expanded SnRK2 families with ABA-responsive subclass III members	Stomatal, transcriptional, and osmotic responses under drought and salinity	Genome-wide analyses and functional studies across selected members	Leaf surface, nodulation, and rhizosphere interactions may alter carrier performance	[25]
Tomato and horticultural eudico	Conserved PYR/PYL–PP2C–SnRK2 signaling	Stomatal regulation, fruit development, and stress recovery	Molecular physiology and nanocarrier uptake studies	Useful for foliar delivery, edible-tissue residue, and fruit-quality validation	[26,80]

**Table 3:** Minimum validation standards for MOF-mediated ABA delivery under drought.

Experimental Component	Minimum Acceptable Evidence	Strong Mechanistic Evidence	Essential Control/Common Failure
Framework identity	Crystallinity, composition, size distribution, charge, and stability	Batch reproducibility plus transformation under application and plant conditions [22]	Do not infer identity from synthesis recipe alone
ABA loading	Loading efficiency and retained ABA activity	Complete mass balance, host–guest interaction evidence, and stereochemical integrity [18,21]	Report both loaded and unbound ABA
Release kinetics	Cumulative release in relevant pH and ionic ranges	Release in simulated apoplast or xylem sap, plant extracts, and intact tissues [21]	Distilled-water release alone is inadequate
Stress-trigger validation	Measured trigger in the intended crop tissue	Spatially and temporally resolved trigger linked quantitatively to release [21,50]	Do not assume all crops reach the same apoplastic pH or redox state
Uptake and localization	Surface washing plus tissue concentration	Confocal or electron microscopy, elemental mapping, and spectroscopy or particle-specific analysis to establish spatial localization, chemical state, and carrier integrity [77,78,89]	Include free-dye, unlabeled-carrier and autofluorescence controls, and confirm label stability to prevent false interpretation of carrier uptake
ABA source	Tissue ABA after treatment	Stable-isotope-labeled ABA separating delivered, endogenous, conjugated, and catabolic pools [12,31]	Total ABA cannot establish delivery mechanism
Carrier contribution	Empty-MOF comparison	Empty MOF, free metal, free linker, physical mixture, and non-responsive carrier [21,43]	Essential-nutrient nodes are not biologically inert
SnRK2 mechanism	Downstream ABA-responsive phenotype	SnRK2 phosphorylation or kinase activity, genetic dependence, and downstream target phosphorylation [63,64]	Transcript abundance does not demonstrate kinase activation
Stomatal response	Aperture or conductance	Time-resolved gas exchange, thermal imaging, ion-channel or guard-cell measurements [27,68]	Detached-leaf assays alone limit whole-plant inference
Hydraulics and photosynthesis	Relative water content and chlorophyll fluorescence	Leaf water potential, hydraulic conductance, gas exchange, PSI/PSII performance, and recovery [69,70]	Single endpoint measurements cannot resolve trade-offs
Agronomic relevance	Biomass under one drought episode	Repeated drought–rewatering cycles, reproductive traits, yield, and water productivity [91]	Seedling survival does not establish yield protection
Safety and fate	Visible phytotoxicity and total tissue metal	Metal speciation, linker products, intact-particle evidence, microbiome effects, edible-tissue residues, and life-cycle analysis [35,87]	Absence of visible injury is not a safety assessment

## Data Availability

This manuscript is a review article and does not report primary data. All data referenced herein are available from the cited published sources.

## Abbreviations

ABA, abscisic acid; ABA^−^, deprotonated anionic ABA; ABA-GE, abscisic acid glucose ester; ABA-H, protonated neutral ABA; ABF, ABA-responsive element-binding factor; ABI5, ABA-insensitive 5; ABCG25/ABCG40, ATP-binding cassette subfamily G transporters 25 and 40; AREB, ABA-responsive element-binding protein; bZIP, basic leucine zipper; BTC, benzene-1,3,5-tricarboxylate; CIPK, calcineurin B-like protein-interacting protein kinase; CYP707A, cytochrome P450 family 707 subfamily A ABA 8′-hydroxylase; MAPKKK, mitogen-activated protein kinase kinase kinase; MIL, Matériaux de l’Institut Lavoisier; MOF, metal–organic framework; NPF, nitrate transporter 1/peptide transporter family; OST1, open stomata 1; PCN, porous coordination network; PP2C, protein phosphatase type 2C; PYR/PYL/RCAR, pyrabactin resistance 1/PYR1-like/regulatory component of ABA–receptor; RAF, RAF-like MAPKKK; RBOHD/RBOHF, respiratory burst oxidase homologs D and F; ROS, reactive oxygen species; SAPK, stress-activated protein kinase; SLAC1, slow anion channel-associated 1; SnRK2, sucrose non-fermenting 1-related protein kinase 2; TOR, target of rapamycin; UiO, University of Oslo framework; UV, ultraviolet; VPD, vapor-pressure deficit; WUE, water-use efficiency; ZIF, zeolitic imidazolate framework.
